# Studies in the Psychology of Sex

**Published:** 1901-12

**Authors:** 


**Studies in the Psychology of Sex.** By HAVELOCK ELLIS. Pp. xi., 276. Philadelphia: F. A. Davis Company. 1901.

The first section of this book is concerned with modesty, and the author has succeeded in giving an admirable exposition of the origin, nature, development and influence of this sense. He regards it as the chief secondary sexual element in woman on the psychical side, lacking which she fails in attractiveness to the average and normal man. The two primary factors in modesty, he considers to be the sexual and social; the former involved in the defensive attitude of the female in sexual relationships (itself dependent on periodicity of function), the latter consequent on the attempts to avoid creating disgust by public performance of acts considered useless or disagreeable. Mr. Ellis shows that modesty is in its origin independent of clothing, anatomical modesty succeeding to physiological. When the danger of offensive action by the male no longer exists, clothing is adopted as an attitude of defence against his eyes. Yet further displacing ideas of disgust, it eventually becomes a means for heightening attraction; the civilised European woman, naked in the presence of others, feels mainly ashamed because revealing herself deprived of her weapons of seduction. This segment of the book is full of interesting matter. The second section deals with phenomena of sexual periodicity; it would be impossible to epitomise the views expressed in this place. The last chapter, on auto-erotism, contains a very valuable discussion on masturbation: the author has treated this important subject in the most capable and dispassionate manner. He concludes that the practice of the habit is not harmful unless carried to excess, and that it is seldom an excitant of insanity except in the presence of heredity. Also excellent is the treatment of the relationship between hysteria and sexual emotion. He indicates the distinction between the response of the psychic sexual organism in the merely hysteroid state and in the morbid condition alone properly termed hysteria; this last may be regarded, in very many cases at least, as a manifestation of the sexual emotions—in other words, a transformation of auto-erotism. Mr. Ellis has shown, in the construction of his book, the thoroughness in research, clearness and delicacy of expression which we are accustomed to expect from him. We trust that the work may receive cordial recognition by those to whom it is addressed; at the same time we hope that every effort will be made by the publishers to limit the issue to thoroughly responsible persons, as appears their intention.

